# Pesticide exposure and cognitive decline in a rural South Korean population

**DOI:** 10.1371/journal.pone.0213738

**Published:** 2019-03-21

**Authors:** Jae-Yeop Kim, Sung-jin Park, Sung-Kyung Kim, Chang-Soo Kim, Tae-Hei Kim, Seong-Ho Min, Sung-Soo Oh, Sang-Baek Koh

**Affiliations:** 1 Department of Occupational and Environmental Medicine, Wonju Severance Christian Hospital, Wonju, Korea; 2 Department of Preventive Medicine and Institute of Occupational and Environmental Medicine, Wonju College of Medicine, Yonsei University, Wonju, Korea; 3 Yonsei University Graduate School, Seoul, Korea; 4 Department of Occupational and Environmental Medicine, Hongseong Medical Center, Hongseong, Korea; 5 Department of Preventive Medicine, Yonsei University College of Medicine, Seoul, Korea; 6 Institute of Human Complexity and Systems Science, Yonsei University, Seoul, Korea; 7 Department of Psychiatry, Wonju Severance Christian Hospital, Wonju, Korea; 8 Institute of Genomic Cohort, Yonsei University Wonju College of Medicine, Wonju, Korea; Nathan S Kline Institute, UNITED STATES

## Abstract

We aimed to investigate the relationship between pesticide exposure and cognitive decline in a rural South Korean population. From July 2015 to December 2017, 200 randomly selected Korean Farmers Cohort study participants were recruited and of these, 169 participants were analyzed. Pesticide exposure was investigated using a standardized questionnaire, and the Korean-Montreal Cognitive Assessment (K-MoCA) was conducted. Cognitive decline was more frequent among those directly exposed to pesticides (*P* = 0.057). Pesticide exposure and cognitive decline were positively correlated in the group with direct exposure versus no exposure (crude odds ratio [OR], 2.66; 95% confidence interval [CI], 1.17–6.04); this relationship was insignificant after adjustment (adjusted OR, 1.50; 95% CI, 0.57–3.92). There was a significant difference in the K-MoCA scores for each group based on pesticide exposure (*P* = 0.003). When we stratified by age, differences in the K-MoCA scores depending on the degree of pesticide exposure in the those aged 60 to 69 years were identified. Overall, there was a tendency towards an association between pesticide exposure and cognitive decline in rural Korean adult farmers. In our study, chronic pesticide exposure tended to have a greater impact in certain age group (60–69 years) than in those under 60 and over 70.

## Introduction

Pesticides are one of several toxic substances intentionally released into the environment to kill living organisms. For example, weeds, insects, fungus and rodents are killed by herbicides, insecticides, fungicides and rodenticides, respectively. Pesticides are also used to prevent diseases, such as malaria and dengue fever, caused by insects and to remove weeds from parks and gardens [1]. Pesticide exposure is associated with diseases such as cancer, hormonal imbalances, asthma, allergies, and hypertension [2]. There is also a series of evidence suggesting that it is also associated with deformities, low birth weight, and abortions [3–4].

The correlation between pesticides use (e.g. for agricultural purposes) and psychiatric diseases has been studied in detail [5–9]. One study showed that agricultural workers have a higher rate of pesticide exposure and mental disorders [6]. In a cross-sectional study of 869 inhabitants of tobacco farms in Brazil, the prevalence of general mental illness and depression was higher in the pesticide exposed group than in the unexposed group [7]. In another cross-sectional study, there was no correlation between exposure to pesticides, and depression and dementia, but there was a correlation with positive screening results for Parkinson's disease and neuropathy [8]. Additionally, another study showed that there was increased risk of developing dementia or Alzheimer's disease among farmers [9]. However, there is still a debate regarding which specific chemicals are linked to the onset of disease, as well as the intensity and type of exposure.

Several studies also showed that chronic pesticide exposure has a variety of neurobehavioral effects, including the onset of Alzheimer's disease and dementia. In a study of 614 farm workers in France, the group with greater exposure to pesticides had a lower score on the cognitive function test than the group with no exposure [3]. In a cross-sectional study conducted in Chile in 2017, the proportion of participants who did not reach the normal threshold for the neurological examination in the exposed group was high [10]. However, epidemiological evidence on the neurobehavioral effects of chronic pesticide exposure are still limited [1], and further research is needed on the neurobehavioral effects of pesticide exposure.

In Korea, residents and farmers in rural areas are subject to chronic pesticide exposure. The annual use of pesticides in Korea increased between the 1970s and 1990s, however since 2001 the annual usage has been decreasing [11]. In 2011, the annual use of pesticides was 19,131 tons; 34.7%, 28.0%, and 27.1% were insecticides, fungicides, and herbicides, respectively [11]. Additionally, most of the pesticides used to date in Korea are pyrethroids and organophosphates [12]. The immediate effects of exposure to high concentrations of organophosphorus pesticides have been well documented as cholinergic crisis. Exposure leads to the inhibition of acetylcholinesterase (AChE), affecting the function of the central, peripheral, and autonomic nervous system [13]. Pyrethroid pesticides, which are generally synthetic pesticides, are used worldwide to control pests in agricultural and residential areas [14] and there is a study that pyrethroid have neurobehavioral effects [15].

There are several studies that have shown a positive correlation between exposure to pesticides and depression in the Korean population [12,16–17]. However, studies on the relationship between neurobehavioral symptoms, such as cognitive decline and pesticide exposure, are limited in Korea. Therefore, we conducted a study on the relationship between pesticide exposure and cognitive decline in rural areas in Korea.

## Materials and methods

### Study population

A survey of pesticide exposures was conducted among 3162 participants from 2005 to 2008 enrolled in the Korea Farmers Cohort study [16]. The participants in this study were recruited from the residents of the Wonju and Pyengchang areas in Gangwon-do Province of the Republic of Korea, who were farmers or agribusinessmen. To recruit participants for the survey, the follow-up steps of the survey were explained in village offices and community health centers in the areas where the survey was to be conducted. We contacted the participants face-to-face, not through email or phone. The survey was also promoted among the community leaders and farmers during leadership meetings, to the Rural Development Administration of Korea, and volunteers registered to participate in the survey.

Between July 2015 and December 2017, we randomly selected 200 Korean Farmers Cohort study participants for enrolment in our study. All participants visited Wonju Severance Christian Hospital and signed their written informed consent in this study. They were asked to complete data collection activities and physical examination through a one-on-one interview with a professional investigator. Thirty-one individuals had missing data for pesticide exposures (data from 2005 to 2008); thus, a total of 169 participants were included. This study was approved by the Institutional Review Board (IRB) of Wonju Christian Hospital (IRB number: CR315018).

### Covariates

Age was treated as a continuous variable and was categorized into three groups (<60, 60–69, and ≥70 years). If participants smoked less than five packs of cigarettes in their lifetime, they were classified as non-smokers and all participants who were classified as smokers had at least 2 years of smoking history. Alcohol use was categorized into two groups based on current alcohol consumption. Current alcohol consumption was divided into current drinking and non–drinking; the non-drinking group included those who drank alcohol in the past but who did not currently drink alcohol. For those in the current drinking group, the duration of drinking was at least 3 years. Educational level was categorized into two groups (middle school or below, high school or above). Body Mass Index (BMI) was treated as a continuous variable and was categorized into two groups (<25, ≥25 kg/m^2^).

### Pesticide exposure assessment

Pesticide exposure was assessed and indirectly estimated using standardized modified questionnaires developed by the Agricultural Health Study [18]. Activity type (e.g., mixing), application method (e.g., hand spraying), use of personal protective equipment (PPE) (e.g., gloves), and personal work habits and hygiene (e.g., taking a bath after using pesticides) were investigated and used for pesticide exposure assessment.

The following five methods were used to assess pesticide exposure. First, if the participants’ occupation was farming, we assumed s/he was exposed to pesticides. Second, the participants were asked to self-report if they used pesticides. Third and fourth, the intensity and cumulative exposure index (CEI) levels of pesticide use were assessed and lower and higher groups were classified based on the 50% percentile. Intensity and CEI levels were calculated using the following equation:

Intensity level = mixing status + application method + equipment repair status × PPE

CEI level = intensity level × spraying year × spraying days per year

The use of Intensity and CEI levels to assess pesticide exposure has been used in other studies [16,19]. Finally, complex exposures for intensity and CEI levels were categorized into three groups based on the following criteria: 81 non-pesticide users were defined as the unexposed group; 60 participants with higher intensity or CEI levels were defined as the direct exposure group; the 28 participants remaining were classified as the indirect exposure group.

### Cognitive decline assessment

Measurements for cognitive decline were made using the K-MoCA. K-MoCA is the Korean version of MoCA and assesses a variety of cognitive domains, such as attention (forward digit span and trail-making test A), memory (20-minute delayed recall using the Seoul Verbal Learning Test and Rey Complex Figure Test), language function (Korean version of the Boston Naming Test and similarity test of Wechsler Adult Intelligence Scale-Fourth Edition), visuospatial ability (copying the Rey Complex Figure Test and clock copying), executive function (semantic fluency for animal using Controlled Oral Word Association Test and clock drawing test), conceptual thinking and orientation [20]. The implementation time of the K-MoCA is approximately 10 to 15 minutes, and the peak score is 30 points. The K-MoCA is also known to be a useful screening tool for cognitive decline in elderly individuals [21]. In our study, we determined that a participant had cognitive decline when the K-MoCA score was less than 23 points.

### Statistical analysis

Differences between the incidence of cognitive decline and demographic and exposure characteristics for each group were assessed using chi-squared tests. Mann-Whitney U test and Kruskal-Wallis test with post hoc Bonferroni tests were conducted to determine if there were differences in the mean and median K-MoCA scores in each group based on pesticide exposure levels. Additionally, a box plot was constructed with K-MoCA scores and the complex exposure level which was stratified by age. A multivariable logistic regression analysis was performed to analyze the relationship between exposure to pesticides and cognitive decline, and sex, age, BMI, smoking status, drinking status, and educational level were adjusted.

All analyses were conducted using IBM SPSS 23.0 (Chicago, IL, USA) and the **R** statistical package, version 3.5.1 (www.r-project.org/). *P*-values less than 0.05 were considered statistically significant.

## Results

Of the 169 enrolled study participants, 128 (75.7%) were males, and the age range was 51–81 years (mean±standard deviation, 66.85±8.02) (Table 1). 92 (54.4%) participants smoked more than five packs of cigarettes during their lifetime and 114 (67.4%) participants were current alcohol consumers. There was a significant increase in the number of people with cognitive decline among those in the higher age and the lower educational level groups. There was no difference in cognitive decline incidence based on sex, body mass index (BMI), smoking status, and alcohol use.

**Table 1:** Demographic characteristics of the study population.

Total (n = 169)	K-MoCA score		*P*
	≥23 (n, %)	<23 (n, %)	
**Sex**			0.460
Male	97 (75.8)	31 (24.2)	
Female	34 (82.9)	7 (17.1)	
**Age (years)**			0.001*
<60	41 (95.3)	2 (4.7)	
60–69	45 (78.9)	12 (21.1)	
≥70	45 (65.2)	24 (34.8)	
**BMI (kg/m**^**2**^**)**			0.476
<25	72 (75.0)	24 (25.0)	
≥25	59 (80.8)	14 (19.2)	
**Smoking status**			0.661
<5 packs during lifetime	58 (75.3)	19 (24.7)	
≥5 packs during lifetime	73 (79.3)	19 (20.7)	
**Alcohol use**			0.733
No	44 (80.0)	11 (20.0)	
Yes	87 (76.3)	27 (23.7)	
**Educational level**			0.011*
Middle school or below	71 (70.3)	30 (29.7)	
High school or above	60 (88.2)	8 (11.8)	

Abbreviations: BMI, Body Mass Index; K-MoCA, Korean-Montreal Cognitive Assessment

*P < 0.05.

The greater the pesticide exposure levels, the more frequently cognitive decline occurred; however, this association was not statistically significant except for exposure assessment based on farmers and pesticide use (Table 2). There was a statistically significant difference in the median of the K-MoCA scores between the pesticide exposed group and the unexposed group (Table 3). However, there was no significant difference between lower exposure and higher exposure groups when the Bonferroni method was implemented.

**Table 2:** Exposure characteristics of the study population.

Total (n = 169)	K-MoCA score		*P*
	≥23 (n, %)	<23 (n, %)	
**Farmer**			0.041*
No	61 (85.9)	10 (14.1)	
Yes	70 (71.4)	28 (28.6)	
**Pesticide Use**			0.035*
No	69 (85.2)	12 (14.8)	
Yes	62 (70.5)	26 (29.5)	
**Intensity Level of Pesticide Use**			0.053
0	69 (85.2)	12 (14.8)	
Lower group	29 (74.4)	10 (25.6)	
Higher group	33 (67.3)	16 (32.7)	
**Cumulative Exposure Index of Pesticide Use**			0.072
0	69 (85.2)	12 (14.8)	
Lower group	29 (70.7)	12 (29.3)	
Higher group	33 (70.2)	14 (29.8)	
**Complex Exposure of Pesticide Use**			0.057
None	69 (85.2)	12 (14.8)	
Indirect exposure	21 (75.0)	7 (25.0)	
Direct exposure	41 (68.3)	19 (31.7)	

Abbreviations: K-MoCA, Korean-Montreal Cognitive Assessment

*P < 0.05

**Table 3:** Mean and median K-MoCA scores with pesticide exposure level.

Total (n = 169)	K-MoCA score Mean±SD, Median (1^st^ quartile– 3^rd^ quartile)	*P*
**Pesticide Use**		<0.001*
No	25.5±3.49, 26.0 (24.0–28.0)	
Yes	24.1±3.28, 24.0 (22.0–27.0)	
**Intensity Level of Pesticide Use**		0.003**
None	25.5±3.49, 26.0 (24.0–28.0)	
Lower group	24.1±2.87, 24.0 (22.5–26.0)	
Higher group	24.0±3.60, 25.0 (22.0–27.0)	
**Cumulative Exposure Index of Pesticide Use**		0.003***
None	25.5±3.49, 26.0 (24.0–28.0)	
Lower group	24.1±2.78, 24.0 (22.0–26.0)	
Higher group	24.0±3.69, 25.0 (22.0–27.0)	
**Complex Exposure of Pesticide Use**		0.003†
None	25.5±3.49. 26.0 (24.0–28.0)	
Indirect exposure	24.4±2.60, 24.5 (22.8–26.3)	
Direct exposure	23.9±3.56, 24.0 (22.0–27.0)	

Abbreviations: SD, standard deviation; K-MoCA, Korean-Montreal Cognitive Assessment

*Mann-Whitney *U* test

**post-hoc p-value, p-values adjusted with the Bonferroni method

None vs Lower group: 0.011

None vs Higher group: 0.021

***post-hoc p-value, p-values adjusted with the Bonferroni method

None vs Lower group: 0.009

None vs Higher group: 0.026

†post-hoc p-value, p-values adjusted with the Bonferroni method

None vs Indirect exposure: 0.085

None vs Direct exposure: 0.004

**Fig 1:**
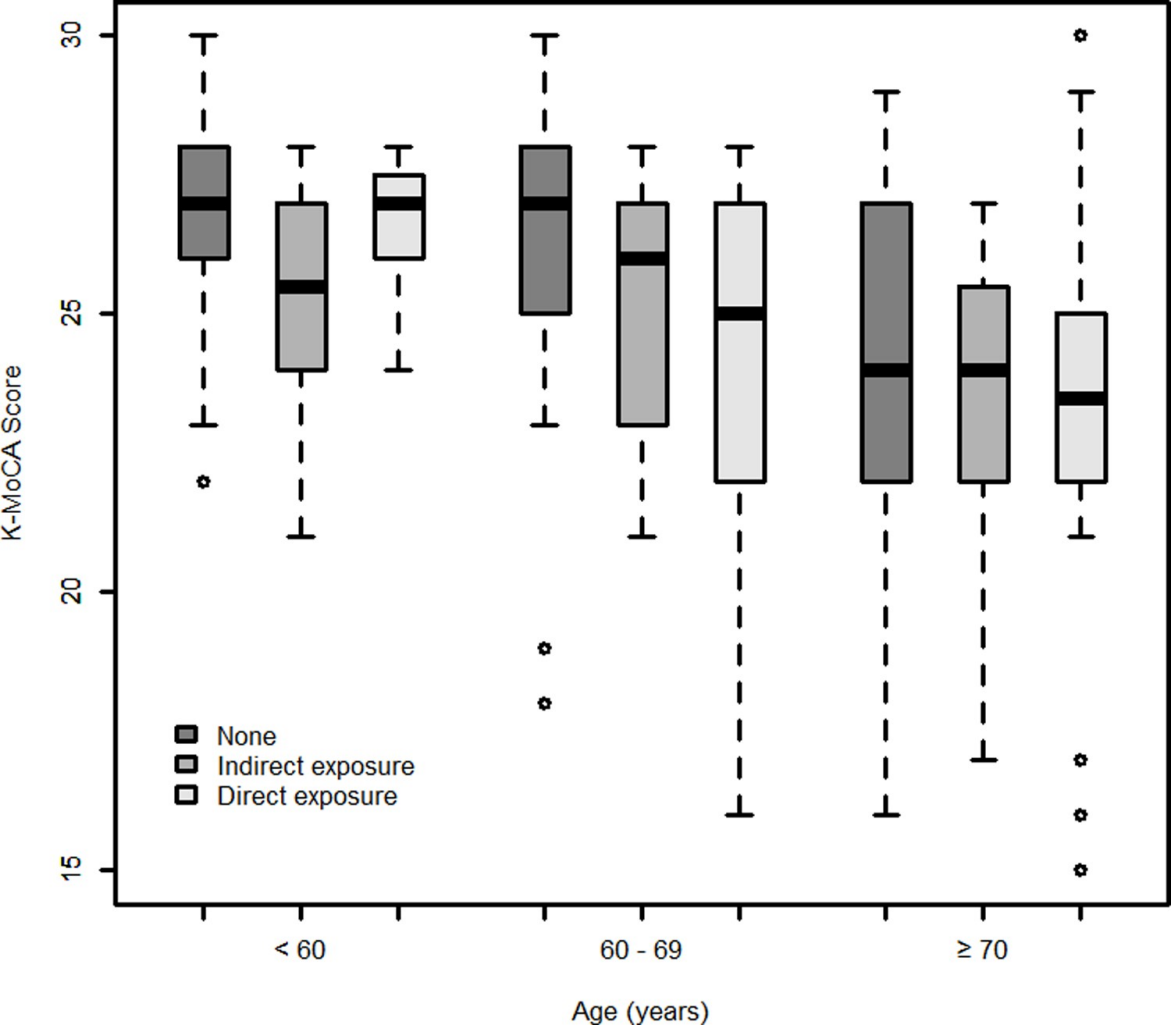
Box plot of K-MoCA score.

Additionally, when stratified by age (Fig 1), there was no significant difference in the K-MoCA scores of pesticide exposure levels in the groups younger than 60 years and older than 70 years. However, in the group between 60 and 69 years, there were differences in K-MoCA score depending on the degree of pesticide exposure (*P* = 0.073).

Upon univariate logistic regression analysis (Table 4), we determined that there was a significant association between pesticide exposure levels and cognitive decline among farmers (crude OR 2.44, 95% CI 1.09–5.42). those who used pesticides (crude OR 2.41, 95% CI 1.12–5.18), higher intensity level group (crude OR 2.78, 95% CI 1.18–6.56), higher CEI level group (crude OR 2.43, 95% CI 1.01–5.85), and direct exposure group (crude OR 2.66, 95% CI 1.17–6.04). However, these relationships were no longer significant after adjustment for potential confounders (farmers adjusted OR [aOR] 1.49, 95% CI 0.59–3.78; pesticide use aOR 1.51, 95% CI 0.62–3.68; higher intensity levels aOR 1.73, 95% CI 0.65–4.65; higher CEI level aOR 1.38, 95% CI 0.49–3.85; direct exposure aOR 1.50, 95% CI 0.57–3.92).

**Table 4:** Logistic regression analysis for pesticide exposure and cognitive decline.

	Cognitive decline (K-MoCA <23)	
Total (n = 169)	**Crude**	**Adjusted†**
**Farmer**		
No	(ref)	(ref)
Yes	2.44 (1.09–5.42)*	1.49 (0.59–3.78)
**Pesticide Use**		
No	(ref)	(ref)
Yes	2.41(1.12–5.18)*	1.51 (0.62–3.68)
**Intensity level of Pesticide Use**		
None	(ref)	(ref)
Lower group	1.98 (0.77–5.10)	1.26 (0.42–3.70)
Higher group	2.78 (1.18–6.56)*	1.73 (0.65–4.65)
**Cumulative Exposure Index of Pesticide Use**		
None	(ref)	(ref)
Lower group	2.37 (0.95–5.91)	1.66 (0.60–4.60)
Higher group	2.43 (1.01–5.85)*	1.38 (0.49–3.85)
**Complex Exposure of Pesticide Use**		
None	(ref)	(ref)
Indirect exposure	1.91 (0.66–5.49)	1.54 (0.48–4.94)
Direct exposure	2.66 (1.17–6.04)*	1.50 (0.57–3.92)

Data presented as odds ratios and 95% confidence intervals.

Abbreviations: K-MoCA, Korean-Montreal Cognitive Assessment

†Adjusted for sex, age, BMI, smoking status, alcohol use, educational level

*P < 0.05

## Discussion

Of the 169 participants included, 38 participants (22.4%) had a K-MoCA score below 23. Cognitive decline incidence was increased for those who were older and had lower educational levels. The group with greater exposure to pesticides tended to have more frequent cognitive decline, but this relationship was not statistically significant. The relationship between exposure to pesticides and cognitive decline was significant before adjustment; however, after adjustment, it was no longer statistically significant.

Several studies have reported that those with chronic pesticide exposure had lower scores on neurological examinations than those who were unexposed [3,11]. Additionally, a meta-analysis was conducted in 2014 to investigate occupational exposure to pesticides and neurobehavioral effects and found that with the exception of one study in adults, there was a significant impact on cognitive function and exercise behavior in most studies [22]. In a meta-analysis of seven studies on Alzheimer's disease and pesticide exposure, there was a correlation between Alzheimer's disease and pesticide exposure; a subgroup analysis showed a better association with higher quality studies [23]. In a systematic review of neurological diseases related to pesticide exposure in 2016, the odd ratios of the two studies related to Alzheimer's disease were 2.39 and 4.35, respectively; however, the data quality was low [24]. Despite the availability of studies that have linked pesticide exposure and cognitive impairment or Alzheimer's disease, epidemiological evidence is still limited.

There is debate regarding exactly which pesticide substances are related to cognitive decline. Studies have shown a positive association between long-term exposure to low doses of paraquat, maneb, dieldrin, pyrethroids, and organophosphorus pesticides and Alzheimer's disease [25]. Additionally, repetitive organophosphorus pesticide exposure can impair attention, memory, and cognitive function by acting on axonal transport, neurotrophins and their receptors, and mitochondria, in addition to causing a "cholinergic crisis" [26]. Some studies have suggested that organophosphorus pesticides play an important role in neurobehavioral and neuropsychological disorders [27].

However, unlike the previous studies, a study of 40 farmers exposed to organophosphorus pesticides and 40 unexposed controls showed inhibition of oxidative stress and AChE, but there was no change in cognitive function [28]. It was also determined that exposure to organophosphorus and pyrethroid pesticides was not associated with cognitive decline and organochlorine pesticides were associated with decreased cognitive function [29]. In a study conducted in the United States among 644 subjects, there was a decrease in cognitive function among those with higher organochlorine concentrations in the body [30]. In Sweden, three organochlorine pesticide concentrations in the body were measured among 989 adults greater than 70 years of age; the risk of cognitive impairment was three times higher in the group with higher concentrations than the group with lower concentrations [31].

Our results showed that there was a tendency for an association between pesticide exposure and cognitive decline; however, this association was not statistically significant. Prior to adjustment, there was a correlation between exposure to pesticides and cognitive decline; however, after adjustment this association was no longer significant. Our findings were consistent with those from previous studies [28,29] and this may have been due to the use of organic phosphorus, pyrethroid pesticides in Korea [12]. Additionally, K-MoCA scores are known to be lower for elderly individuals and those with lower educational levels. In our study, the participants in the direct exposure group had lower educational levels and were older than those in the unexposed group, which may be the cause of the confounding or interaction noted in our study. Overall, 30.9% of participants in the unexposed group were greater than 70 years of age, while 42.7% and 53.3% of those in the indirect and direct exposure groups were greater than 70 years of age. Also, 49.4% of participants in the unexposed group had low educational levels, compared to 57.1% and 75.0% of participants in the indirect and direct exposure groups. Finally, the small number of participants enrolled may have impacted this tendency.

K-MoCA has an excellent sensitivity of 89% and a good specificity of 84% for screening for mild cognitive impairment (MCI) and its internal consistency (Cronbach’s alpha was 0.86) and test-retest reliability (Intraclass correlation coefficients [ICCs] = 0.74, p-value < 0.001) are also good [21]. Additionally, K-MoCA is a test which can identify MCI regardless of education levels [32]. However, in a study of the MoCA test among 2653 participants from the general population, 66% of participants did not meet the 26-point cutoff value based on the previous data, and the authors suggested caution when applying the recommended cut scores [33]. In our study, we have defined a cognitive decline for a particular cutoff value (<23), regardless of age and education level, so there could be errors. When the K-MoCA score was considered as a continuous variable, the mean K-MoCA score in the exposed group was significantly lower than in the non-exposed group.

When we stratified by age (Fig 1), there were differences in the K-MoCA scores depending on the degree of pesticide exposure in the group between 60 and 69 years. However, in the groups younger than 60 years and older than 70 years, there were no differences in the K-MoCA scores. The authors suggested that these results could support the new hypothesis that cognitive decline occurs more rapidly in the chronic and direct pesticide exposure group than in the unexposed group. In younger people with normal cognitive function or in older people with greatly reduced cognitive function, chronic pesticide exposure may not have a significant impact. However, chronic pesticide exposure may increase cognitive decline with age. We searched for previous research on whether chronic pesticide exposure affects the degree of cognitive decline with age, but we could not find any relevant studies in the literature.

To our knowledge, this is the first study on the relationship between pesticide exposure and cognitive dysfunction in Korea. Previous studies have investigated the relationship between exposure to pesticides and depression in Korea [12,16–17] and mortality as a result of leukemia in rural areas [34]; however, the studies on the effects of pesticide exposure on humans in Korea is insufficient.

This study was subject to several limitations. First, there was greater than a 10-year difference between the first pesticide exposure survey and the K-MOCA questionnaire survey. There was no accurate investigation of the types of pesticides used nor were there quantitative measurements of biomarkers (e.g. metabolites of organophosphate in urine). Additionally, as a result of the small sample size and the cross-sectional design, our study has limited power and was not able to determine causality. Moreover, the present study was a pilot study; investigators will continue to investigate pesticide exposure and psychiatric parameters among participants in the Korea Farmers Cohort study. In future studies, longitudinal studies should be conducted with adjustment for age and educational levels because they are major confounding factors.

## Conclusion

In some rural areas of Korea, pesticide exposure and cognitive decline showed a tendency to be related; however, this relationship was not statistically significant. However, there was a difference in the mean K-MoCA scores between the pesticide exposure groups. In our study, chronic pesticide exposure tended to have a greater impact in certain age group (60–69 years) than in those under 60 and over 70. Future research should be conducted on pesticide exposure and psychiatric diseases in which there is accurate and quantitative investigation of the chemicals or mixture of the pesticides involved in exposure. Additionally, we think that psychiatric testing should be done in populations with potential pesticide exposure.

## Supporting information

S1 File Pesticide exposure questionnaire. (English)
(DOCX)

S2 File Pesticide exposure questionnaire. (Korean)
(DOCX)

S3 File Data set. (XLSX)
