# Correction: Pesticide exposure and cognitive decline in a rural South Korean population

**DOI:** 10.1371/journal.pone.0216310

**Published:** 2019-04-25

**Authors:** Jae-Yeop Kim, Sungjin Park, Sung-Kyung Kim, Chang-Soo Kim, Tae-Hei Kim, Seong-Ho Min, Sung-Soo Oh, Sang-Baek Koh

The second author’s name is spelled incorrectly. The correct name is: Sungjin Park. The correct citation is: Kim J-Y, Park S, Kim S-K, Kim C-S, Kim T-H, Min S-H, et al. (2019) Pesticide exposure and cognitive decline in a rural South Korean population. PLoS ONE 14(3): e0213738. https://doi.org/10.1371/journal.pone.0213738
