# Supplementary material for: Pesticide exposure and cognitive decline in a rural South Korean population
Source: PLoS One. 2019 Mar 21;14(3):e0213738. doi: 10.1371/journal.pone.0213738 (PMC6428296; doi:10.1371/journal.pone.0213738)
Supplement: S1 File — (English) (DOCX) [file pone.0213738.s001.docx]

| \| The entire number \|  \|  \| \|  \| \|  \| \|  \| \|  \| \|  \| \|  \| \| \| --- \| --- \| --- \| --- \| --- \| --- \| --- \| --- \| --- \| --- \| --- \| --- \| --- \| --- \| --- \| --- \| \| Unique number \|  \| \|  \| \| - \| \|  \|  \|  \| \|  \| \|  \| \| | | | | | | | | | | | | | | | | | | | | | | | |
| --- | --- | --- | --- | --- | --- | --- | --- | --- | --- | --- | --- | --- | --- | --- | --- | --- | --- | --- | --- | --- | --- | --- | --- | --- | --- | --- | --- | --- | --- | --- | --- | --- | --- | --- | --- | --- | --- | --- | --- | --- | --- | --- | --- | --- | --- | --- | --- | --- | --- | --- | --- | --- | --- | --- |
|  | | | | | | | | | | | | | | | | | | | | | | | |
| **Pesticides Exposure and chronic health impact assessment survey** | | | | | | | | | | | | | | | | | | | | | | | |
|  | | | | | | | | | | | | | | | | | | | | | | | |
| **A joint study by the University of the RDA in each region for the chronic poisoning of pesticides health effects survey for evaluating and performing. This survey is the pesticide spraying and related health problems that can determine the cause of the pesticide exposure-related diseases prevention and measures seeking to help build will be conducted.**  **Questionnaire in the form of pesticides usage and spray, etc. with agricultural work and are investigating, pesticides can cause a disease is to confirm the information. The answer to the law on protection of personal information the personal information public institutions such the RDA is managed strictly in future disease occurs, other than for the purpose of verification is not used. Thank you very much for your participation in the survey.**  **1. I responded to my request freely by listening to the explanation of the survey's purpose and process. ( ) Yes, ( ) no**  **2. I agree to confirm the occurrence of the disease by linking public data (National Statistical Office, National Health Insurance Corporation, National Cancer Center, etc.) with the purpose of confirming the occurrence and death of the disease.**  **( ) Yes, ( ) no**  **Participants name _______________, signature _____________** | | | | | | | | | | | | | | | | | | | | | | | |
|  | | | | | | | | | | | | | | | | | | | | | | | |
| **name** |  | | | | | | | | | | | | | | | **gender** | | | | | ****_1_ man ****_0_ woman | | |
| **Resident registration number** |  |  |  | |  | |  |  | | **-** |  | |  | |  |  | |  |  |  | **age** | | years |
| **Contact phone number** |  | | | | | | | | | | | | | | | | | | | | | | |
| **Survey date and time** | **year** | | | | | **month** | | | | | | **day** | | | | | **Investigator's name** | | | | |  | |
|  |  | | |  | | | | |  | | | | |  | | | | | | | |  | |
|  |  | | |  | | | | |  | | | | |  | | | | | | | |  | |
|  |  | | |  | | | | |  | | | | |  | | | | | | | |  | |

| **Part 1. Pesticide exposure** | | | | | | | | |
| --- | --- | --- | --- | --- | --- | --- | --- | --- |
|  | | | | | | | | |
| **1.** I will ask you about the area you live in and the length of your stay. | | | | | | | | |
|  | | | | | | | | |
|  | **Residence period** | | **Residential address** | | | | | |
| Current residence | year month  ~ year month | |  | | | | | |
|  | | | | | | | | |
| **2.** Are you currently farming? | | | | | | | | |
| ****_0_ No, I have never been a farmer.. | | **☞ Part 2. Go to health impact questions.** | | | | |  | |
|  | | | | | | | | |
| ****_1_ It was a farmer in the past, but not now. (**2-1. Total period of farming in the past year**) | | | | | | | | |
| \| **☞ Part 2. Go to health impact questions** \| \| --- \| | | | | | | | | |
| ****_2_ Yes. I am currently doing farming.. (**2-2. What is the age at which agriculture began as a main business?**  , **Total period of farming year** | | | | | |  | | |
|  | | | | | | | | |
| **3.** Are you currently spraying pesticides? | | | | | | | | |
| ****_0_ no. I have never sprayed pesticides at all. | | | | **☞ Part 2. Go to health impact questions.** | | | |  |
| ****_1_ I have sprayed pesticides in the past, but not now. | | | (**3-1. Total period of past pesticide application**  ) | | | | | |
|  | | |  | | **☞ Part 2. Go to health impact questions.** | | | |
|  | | | | | | | | |
| ****_2_ I did not spray the pesticide directly, but I helped the pesticide sprayer. (Pesticide mixture, etc). | | | | | | | | |
| ****_3_ Yes. We are currently spraying pesticides directly. | | | | | | | | |
| **3-2. How many years did you spray the pesticide?** year | | | | | | | | |
| **3-3. Did you spray the pesticide on average for a few days each year?** Annual days | | | | | | | | |
| **3-4. On average, how many hours did you spray the pesticide application day?** Daily hours | | | | | | | | |
| **3-5.** When using pesticides, do you use more than two pesticides? | | | | | | | | |
| ****_0_ No. | | | | | | | | |
| ****_1_ Yes. (**3-6. How many kinds of pesticides did you mix?** ) | | | | | | | | |
| **3-7**. **How old were you when you first used pesticides?**  years | | | | | | | | |

| **4.** Let me ask you about the individual pesticides that you have used for crops and livestock that you have cultivated professionally over the past year.  Please write down the number of pesticides and spraying methods used by each piece. | | | | | | | | | |
| --- | --- | --- | --- | --- | --- | --- | --- | --- | --- |
|  | **Agricultural products** | **Scale** | **Farming period** | **Annual Spray Number** | **Spray time per unit** | **Spray amount per one time (L or Kg)** | **How to spray (**example **②)** | **When was the first year you used pesticides?** | **How many years did you spray pesticide?** |
|  | rice plant |  |  |  |  |  |  |  |  |
| **Cultivated crops** |  |  |  |  |  |  |  |  |  |
|  |  |  |  |  |  |  |  |  |  |
|  |  |  |  |  |  |  |  |  |  |
| **fruit tree** |  |  |  |  |  |  |  |  |  |
|  |  |  |  |  |  |  |  |  |  |
|  |  |  |  |  |  |  |  |  |  |
| **Facility-grown crops** |  |  |  |  |  |  |  |  |  |
|  |  |  |  |  |  |  |  |  |  |
|  |  |  |  |  |  |  |  |  |  |
| **Livestock** |  |  |  |  |  |  |  |  |  |
|  |  |  |  |  |  |  |  |  |  |
|  |  |  |  |  |  |  |  |  |  |
| **How to spray (**example **②)** | | | | | | | | | |
| 1. Hand spray (powered) 2. Hand spray (manual) 3. Backlash type sprayer (powered) 4. Backlash type sprayer (manual type) 5. High Pressure Sprayer (SS) - With cap | | | | | | | | | |
| 6. High Pressure Sprayer (SS) - No Cap 7. Firearm 8. Hand-sprinkled (grain, powder, etc.) 9. Seed disinfection (immersion) 10. 기타 Other (Please specify your method directly in the table.) | | | | | | | | | |

| **5.** **Please indicate the degree of use of each protection box below (✔check).** | | | | | | | | | | | | | | | | | |
| --- | --- | --- | --- | --- | --- | --- | --- | --- | --- | --- | --- | --- | --- | --- | --- | --- | --- |
| **Protective equipment** | | | **Usage frequency** | | | | | | | | | | | | | | |
|  |  |  | **Almost never wear** | | | **Sometimes worn** | | | **Almost worn** | | | | **Always worn** | | | | |
| **(1) Protective apparel** | | | ① | | | ② | | | ③ | | | | ④ | | | | |
| **(2) Under waterproof** | | | ① | | | ② | | | ③ | | | | ④ | | | | |
| **(3) hat** | | | ① | | | ② | | | ③ | | | | ④ | | | | |
| **(4) Gas mask** | | | ① | | | ② | | | ③ | | | | ④ | | | | |
| **(5) goggles** | | | ① | | | ② | | | ③ | | | | ④ | | | | |
| **(6)** **Rubber glove** | | | ① | | | ② | | | ③ | | | | ④ | | | | |
| **(7) Rubber boots** | | | ① | | | ② | | | ③ | | | | ④ | | | | |
| *** No cotton mask, cotton gloves, etc.** | | | | | | | | | | | | | | | | | |
|  | | | | | | | | | | | | | | | | | |
| **6.** **Please indicate compliance with the following items (✔check).** | | | | | | | | | | | | | | | | | |
| **Compliance Statement** | | | | | | | | | | **Compliance Department** | | | | | | | |
|  |  |  |  |  |  |  |  |  |  | **Do not keep** | | **sometimes**  **Keep** | | **generally**  **Keep** | | **Always keep** | |
| **(1) Keep usage and capacity as described** | | | | | | | | | | ① | | ② | | ③ | | ④ | |
| **(2) Wear masks and gloves when diluting pesticides.** | | | | | | | | | | ① | | ② | | ③ | | ④ | |
| **(3) Do not drink or smoke while spraying pesticides** | | | | | | | | | | ① | | ② | | ③ | | ④ | |
| **(4) When I am tired, I do not spray.** | | | | | | | | | | ① | | ② | | ③ | | ④ | |
| **(5) It does not sow at midday.** | | | | | | | | | | ① | | ② | | ③ | | ④ | |
| **(6) After spraying the pesticide, change clothes immediately** | | | | | | | | | | ① | | ② | | ③ | | ④ | |
| **(7) After spraying with pesticides, take a bath with soap.** | | | | | | | | | | ① | | ② | | ③ | | ④ | |
| **(8) The wind piles up when spraying pesticides** | | | | | | | | | | ① | | ② | | ③ | | ④ | |
| **(9) After an hour's work, I will take a break for 10 minutes.** | | | | | | | | | | ① | | ② | | ③ | | ④ | |
| **(10) When repairing or cleaning equipment used for pesticide use, wear mask and gloves Wear** | | | | | | | | | | ① | | ② | | ③ | | ④ | |
| **Part2. health impact** | | | | | | | | | | | | | | | | | |
|  | | | | | | | | | | | | | | | | | |
| Illness | | | | | | | | | | | | | | | | | |
| **1.** If you are suffering from or suffering from a chronic illness listed below, please check the box below. (**✔check**). | | | | | | | | | | | | | | | | | |
|  | | | | | | | | | | | | | | | | | |
|  | **I have ever been sick** | | | **I have been ill for more than 3 months in the last year** | | | **I was diagnosed by a doctor.** | | | **The first time a doctor was diagnosed** | | | | **Current treatment** | | | |
|  | **NO** | | **YES** | **NO** | | **YES** | **NO** | **YES** | |  |  |  |  | **NO** | | **YES** | |
|  |  | |  |  | |  |  |  | |  | | | |  | |  | |
| **A. Circulating system** |  | |  |  | |  |  |  | |  | | | |  | |  | |
| **(1) High blood pressure** | ☐_0_ | | ☐_1_ | ☐_0_ | | ☐_1_ | ☐_0_ | ☐_1_ | |  | | | | ☐_0_ | | ☐_1_ | |
| **(2) Hyperlipidemia** | ☐_0_ | | ☐_1_ | ☐_0_ | | ☐_1_ | ☐_0_ | ☐_1_ | |  | | | | ☐_0_ | | ☐_1_ | |
| **(3) brain stroke** | ☐_0_ | | ☐_1_ | ☐_0_ | | ☐_1_ | ☐_0_ | ☐_1_ | |  | | | | ☐_0_ | | ☐_1_ | |
| **(4)Myocardial infarct** | ☐_0_ | | ☐_1_ | ☐_0_ | | ☐_1_ | ☐_0_ | ☐_1_ | |  | | | | ☐_0_ | | ☐_1_ | |
| **(5) angina pectoris** | ☐_0_ | | ☐_1_ | ☐_0_ | | ☐_1_ | ☐_0_ | ☐_1_ | |  | | | | ☐_0_ | | ☐_1_ | |
|  |  | |  |  | |  |  |  | |  | | | |  | |  | |
|  |  | |  |  | |  |  |  | |  | | | |  | |  | |
| **B. Musculoskeletal system** |  |  |  |  |  |  |  |  |  |  |  |  |  |  |  |  |  |
| **(6) Osteoarthritis** | ☐_0_ | | ☐_1_ | ☐_0_ | | ☐_1_ | ☐_0_ | ☐_1_ | |  | | | | ☐_0_ | | ☐_1_ | |
| **(7) Rheumatoid arthritis** | ☐_0_ | | ☐_1_ | ☐_0_ | | ☐_1_ | ☐_0_ | ☐_1_ | |  | | | | ☐_0_ | | ☐_1_ | |
| **(8) osteoporosis** | ☐_0_ | | ☐_1_ | ☐_0_ | | ☐_1_ | ☐_0_ | ☐_1_ | |  | | | | ☐_0_ | | ☐_1_ | |
| **(9) Herniated disc** | ☐_0_ | | ☐_1_ | ☐_0_ | | ☐_1_ | ☐_0_ | ☐_1_ | |  | | | | ☐_0_ | | ☐_1_ | |
|  | | | | | | | | | | | | | | | | | |
| **C. Respiratory system** | | | | | | | | | | | | | | | | | |
| **(10) pulmonary tuberculosis** | ☐_0_ | | ☐_1_ | ☐_0_ | | ☐_1_ | ☐_0_ | ☐_1_ | |  | | | | ☐_0_ | | ☐_1_ | |
| **(11) non-pulmonary tuberculosis** | ☐_0_ | | ☐_1_ | ☐_0_ | | ☐_1_ | ☐_0_ | ☐_1_ | |  | | | | ☐_0_ | | ☐_1_ | |
| **(12) asthma** | ☐_0_ | | ☐_1_ | ☐_0_ | | ☐_1_ | ☐_0_ | ☐_1_ | |  | | | | ☐_0_ | | ☐_1_ | |
| **(13) Chronic obstructive lung disease** | ☐_0_ | | ☐_1_ | ☐_0_ | | ☐_1_ | ☐_0_ | ☐_1_ | |  | | | | ☐_0_ | | ☐_1_ | |
| **(14) Sinusitis** | ☐_0_ | | ☐_1_ | ☐_0_ | | ☐_1_ | ☐_0_ | ☐_1_ | |  | | | | ☐_0_ | | ☐_1_ | |
| **(15) Bronchiectasis** | ☐_0_ | | ☐_1_ | ☐_0_ | | ☐_1_ | ☐_0_ | ☐_1_ | |  | | | | ☐_0_ | | ☐_1_ | |
| **(16) Allergic rhinitis** | ☐_0_ | | ☐_1_ | ☐_0_ | | ☐_1_ | ☐_0_ | ☐_1_ | |  | | | | ☐_0_ | | ☐_1_ | |
|  | | | | | | | | | | | | | | | | | |
| **D. Endocrine Disease** | | | | | | | | | | | | | | | | | |
| **(17) diabetes** | ☐_0_ | | ☐_1_ | ☐_0_ | | ☐_1_ | ☐_0_ | ☐_1_ | |  | | | | ☐_0_ | | ☐_1_ | |
| **(18) Thyroid disorder** | ☐_0_ | | ☐_1_ | ☐_0_ | | ☐_1_ | ☐_0_ | ☐_1_ | |  | | | | ☐_0_ | | ☐_1_ | |

|  | | **I have ever been sick** | | | **I have been ill for more than 3 months in the last year** | | | **I was diagnosed by a doctor.** | | **The first time a doctor was diagnosed** | **Current treatment** | |
| --- | --- | --- | --- | --- | --- | --- | --- | --- | --- | --- | --- | --- |
|  |  | **NO** | | **YES** | **NO** | **YES** | | **NO** | **YES** |  | **NO** | **YES** |
|  | | | | | | | | | | | | |
| **E. Eye, Ear disease** | | | | | | | | | | | | |
| **(19) Cataract** | | ☐_0_ | | ☐_1_ | ☐_0_ | ☐_1_ | | ☐_0_ | ☐_1_ |  | ☐_0_ | ☐_1_ |
| **(20) glaucoma** | | ☐_0_ | | ☐_1_ | ☐_0_ | ☐_1_ | | ☐_0_ | ☐_1_ |  | ☐_0_ | ☐_1_ |
| **(21) Retinal degeneration** | | ☐_0_ | | ☐_1_ | ☐_0_ | ☐_1_ | | ☐_0_ | ☐_1_ |  | ☐_0_ | ☐_1_ |
| **F. Cancer** | | | | | | | | | | | | |
| **(22) Gastric cancer** | | ☐_0_ | | ☐_1_ | ☐_0_ | ☐_1_ | | ☐_0_ | ☐_1_ |  | ☐_0_ | ☐_1_ |
| **(23) Hepatic cancer** | | ☐_0_ | | ☐_1_ | ☐_0_ | ☐_1_ | | ☐_0_ | ☐_1_ |  | ☐_0_ | ☐_1_ |
| **(24) Colon cancer** | | ☐_0_ | | ☐_1_ | ☐_0_ | ☐_1_ | | ☐_0_ | ☐_1_ |  | ☐_0_ | ☐_1_ |
| **(25) Breast cancer** | | ☐_0_ | | ☐_1_ | ☐_0_ | ☐_1_ | | ☐_0_ | ☐_1_ |  | ☐_0_ | ☐_1_ |
| **(26) Cervical cancer** | | ☐_0_ | | ☐_1_ | ☐_0_ | ☐_1_ | | ☐_0_ | ☐_1_ |  | ☐_0_ | ☐_1_ |
| **(27) Lung cancer** | | ☐_0_ | | ☐_1_ | ☐_0_ | ☐_1_ | | ☐_0_ | ☐_1_ |  | ☐_0_ | ☐_1_ |
| **(28) other cancer**  **(: )** | | ☐_0_ | | ☐_1_ | ☐_0_ | ☐_1_ | | ☐_0_ | ☐_1_ |  | ☐_0_ | ☐_1_ |
| **(29) other cancer**  **(: )** | | ☐_0_ | | ☐_1_ | ☐_0_ | ☐_1_ | | ☐_0_ | ☐_1_ |  | ☐_0_ | ☐_1_ |
|  | | | | | | | | | | | | |
| **G. Digestive system** | | | | | | | | | | | | |
| **(30) Gastroduodenal ulcer** | | ☐_0_ | | ☐_1_ | ☐_0_ | ☐_1_ | | ☐_0_ | ☐_1_ |  | ☐_0_ | ☐_1_ |
| **(31) Hepatitis B** | | ☐_0_ | | ☐_1_ | ☐_0_ | ☐_1_ | | ☐_0_ | ☐_1_ |  | ☐_0_ | ☐_1_ |
| **(32) Hepatitis C** | | ☐_0_ | | ☐_1_ | ☐_0_ | ☐_1_ | | ☐_0_ | ☐_1_ |  | ☐_0_ | ☐_1_ |
| **(33) Liver Cirrhosis** | | ☐_0_ | | ☐_1_ | ☐_0_ | ☐_1_ | | ☐_0_ | ☐_1_ |  | ☐_0_ | ☐_1_ |
|  | | | | | | | | | | | | |
| **H. Other diseases** | | | | | | | | | | | | |
| **(34) depression** | | ☐_0_ | | ☐_1_ | ☐_0_ | ☐_1_ | | ☐_0_ | ☐_1_ |  | ☐_0_ | ☐_1_ |
| **(35) Atopic dermatitis** | | ☐_0_ | | ☐_1_ | ☐_0_ | ☐_1_ | | ☐_0_ | ☐_1_ |  | ☐_0_ | ☐_1_ |
| **(36) Kidney failure** | | ☐_0_ | | ☐_1_ | ☐_0_ | ☐_1_ | | ☐_0_ | ☐_1_ |  | ☐_0_ | ☐_1_ |
| Family History | | | | | | | | | | | |  |
| **2.** Has your immediate family member (parent, sibling, or child) been diagnosed with or died from the following diseases from your doctor or clinic? Please check if there is, and please indicate how many minutes you have. | | | | | | | | | | | |  |
|  | | | | | | | | | | | |  |
| **Disease name** | | **none** | | | | **Yes (Parents, siblings, children)** | | | | | |  |
| **High blood pressure** | | ☐_0_ | | | | ☐_1_ ( persons) | | | | | |  |
| **diabetes** | | ☐_0_ | | | | ☐_1_ ( persons) | | | | | |  |
| **Angina or myocardial infarction** | | ☐_0_ | | | | ☐_1_ ( persons) | | | | | |  |
| **brain stroke** | | ☐_0_ | | | | ☐_1_ ( persons) | | | | | |  |
| **Gastric cancer** | | ☐_0_ | | | | ☐_1_ ( persons) | | | | | |  |
| **Lung cancer** | | ☐_0_ | | | | ☐_1_ ( persons) | | | | | |  |
| **Breast cancer** | | ☐_0_ | | | | ☐_1_ ( persons) | | | | | |  |
| **Colon cancer** | | ☐_0_ | | | | ☐_1_ ( persons) | | | | | |  |
| **Hepatic cancer** | | ☐_0_ | | | | ☐_1_ ( persons) | | | | | |  |
| **Cervical cancer** | | ☐_0_ | | | | ☐_1_ ( persons) | | | | | |  |
| **Gallbladder cancer** | | ☐_0_ | | | | ☐_1_ ( persons) | | | | | |  |
| **Thyroid cancer** | | ☐_0_ | | | | ☐_1_ ( persons) | | | | | |  |
| **Pancreatic cancer** | | ☐_0_ | | | | ☐_1_ ( persons) | | | | | |  |
| **Prostate cancer** | | ☐_0_ | | | | ☐_1_ ( persons) | | | | | |  |
| **Ovarian cancer** | | ☐_0_ | | | | ☐_1_ ( persons) | | | | | |  |
| **Non-Hodgkin's lymphoma** | | ☐_0_ | | | | ☐_1_ ( persons) | | | | | |  |
| **Pharyngeal cancer** | | ☐_0_ | | | | ☐_1_ ( persons) | | | | | |  |
| **Laryngeal cancer** | | ☐_0_ | | | | ☐_1_ ( persons) | | | | | |  |
| **cutaneous cancer** | | ☐_0_ | | | | ☐_1_ ( persons) | | | | | |  |
| **Testicular cancer** | | ☐_0_ | | | | ☐_1_ ( persons) | | | | | |  |
| **Kidney cancer** | | ☐_0_ | | | | ☐_1_ ( persons) | | | | | |  |
| **Brain and central nervous system cancer** | | ☐_0_ | | | | ☐_1_ ( persons) | | | | | |  |
| **Multiple myeloma** | | ☐_0_ | | | | ☐_1_ ( persons) | | | | | |  |
| **leukemia** | | ☐_0_ | | | | ☐_1_ ( persons) | | | | | |  |
| **Bone marrow cancer** | | ☐_0_ | | | | ☐_1_ ( persons) | | | | | |  |
| **Connective tissue or other soft tissue cancer** | | ☐_0_ | | | | ☐_1_ ( persons) | | | | | |  |
| **Salivary gland cancer** | | ☐_0_ | | | | ☐_1_ ( persons) | | | | | |  |
| **Other malignant tumors ( )** | | ☐_0_ | | | | ☐_1_ ( persons) | | | | | |  |

| Mental Health | | |
| --- | --- | --- |
|  | | |
| **3.** Please include the following questions and mark them in the answer for your present state (✔check). | | |
|  | | |
|  | **No** | **Yes** |
| **(1) Are you generally satisfied with your current life?** | ☐_0_ | ☐_1_ |
| **(2) Have you lost much of your activity or motivation these days?** | ☐_0_ | ☐_1_ |
| **(3) Do you feel you are living in vain?** | ☐_0_ | ☐_1_ |
| **(4) Do you often feel boring when you feel that you are living in vain?** | ☐_0_ | ☐_1_ |
| **(5) Do you usually feel refreshing?** | ☐_0_ | ☐_1_ |
| **(6) Do you feel uneasy because you have an ominous affair?** | ☐_0_ | ☐_1_ |
| **(7) Are you generally happy?** | ☐_0_ | ☐_1_ |
| **(8) Do you often feel desperate?** | ☐_0_ | ☐_1_ |
| **(9) Do not you want to go outside and stay at home?** | ☐_0_ | ☐_1_ |
| **(10) Do you feel that your memory is worse than other elderly people of similar age?** | ☐_0_ | ☐_1_ |
| **(11) Do you enjoy being alive now?** | ☐_0_ | ☐_1_ |
| **(12) Do you feel that I am useless?** | ☐_0_ | ☐_1_ |
| **(13) Are you feeling good?** | ☐_0_ | ☐_1_ |
| **(14) Do you feel that you have no hope at all now?** | ☐_0_ | ☐_1_ |
| **(15) Do you think you are worse than others?** | ☐_0_ | ☐_1_ |
|  | | |
| **3-1. Have you ever thought about wanting to die in the last year?** | | |
| ****_0_ no | | |
| ****_1_ yes | | |
| **3-2. Have you ever actually committed suicide in the last year?** | | |
| ****_0_ no | | |
| ****_1_ yes | | |

| Pesticide poisoning | | | | | | | | |
| --- | --- | --- | --- | --- | --- | --- | --- | --- |
|  | | | | | | | | |
| **4. Have you been addicted to pesticides over the past year?** | | | | | | | | |
| ****_0_ no | | | | | | | | |
| ****_1_ yes | | | | | | | | |
|  | | | | | | | | |
| **5. Please write only those who answered "Yes" to question 4.** | | | | | | | | |
| **Date of occurrence** | **Pesticide name** | **Situation at the time of poisoning ^1)^** | **Intentionality ^2)^** | | **Treatment** | | **Treatment Location ^3)^** | **What was the cost of the treatment?** |
|  |  |  |  | | ****_0_ I was not treated. | |  |  |
|  |  |  |  |  | ****_1_ I received outpatient treatment. (Period of Visit: days | |  |  |
|  |  |  |  |  | ****_2_ I was hospitalized.  (Hospitalization period: days) | |  |  |
|  |  |  |  | | ****_0_ I was not treated. | |  |  |
|  |  |  |  |  | ****_1_ I received outpatient treatment. (Period of Visit: days | |  |  |
|  |  |  |  |  | ****_2_ I was hospitalized. (Hospitalization period: days) | |  |  |
|  |  |  |  | | ****_0_ I was not treated. | |  |  |
|  |  |  |  |  | ****_1_ I received outpatient treatment. (Period of Visit: days | |  |  |
|  |  |  |  |  | ****_2_ I was hospitalized. (Hospitalization period: days) | |  |  |
| **example** | | | | | | | | |
| **1) Situation at the time of poisoning** | | ① After pesticide application | | ② After pesticide mixing and preparation | | ③ Pesticide spraying machine after washing | | |
|  |  | ④ Other situations besides agricultural work ( ) | | | | | | |
| **2) Intentionality** | | ① Working | | ② Attempted suicide | | ③ Other ( ) | | |
| **3)Treatment Location** | | ① Public Health Center | | ② Hospitals and clinics | | ③ Other ( ) | | |
