# Supplementary material for: Pesticide exposure and cognitive decline in a rural South Korean population
Source: PLoS One. 2019 Mar 21;14(3):e0213738. doi: 10.1371/journal.pone.0213738 (PMC6428296; doi:10.1371/journal.pone.0213738)
Supplement: S2 File — (Korean) (DOCX) [file pone.0213738.s002.docx]

| \| 전체번호 \|  \|  \|  \|  \|  \|  \|  \|  \| \| --- \| --- \| --- \| --- \| --- \| --- \| --- \| --- \| --- \|  \| 고유번호 \|  \|  \| - \|  \|  \|  \|  \|  \| \| --- \| --- \| --- \| --- \| --- \| --- \| --- \| --- \| --- \| | | | | | | | | | | | | | | | | | | | | |
| --- | --- | --- | --- | --- | --- | --- | --- | --- | --- | --- | --- | --- | --- | --- | --- | --- | --- | --- | --- | --- | --- | --- | --- | --- | --- | --- | --- | --- | --- | --- | --- | --- | --- | --- | --- | --- | --- | --- |
|  | | | | | | | | | | | | | | | | | | | | |
| **농약 노출과 만성 건강영향 평가 조사** | | | | | | | | | | | | | | | | | | | | |
|  | | | | | | | | | | | | | | | | | | | | |
| **농촌진흥청에서는 각 지역별 대학의 공동연구로 농약의 만성중독에 대한 건강영향을 평가하기 위한 조사를 수행하고 있습니다. 본 조사는 농약 살포와 관련된 건강상의 문제와 그 원인을 파악하여 농약 노출과 관련된 질병을 예방하고 대책을 세우는데 일조하고자 실시하는 것입니다.**  **설문지는 농작업과 농약사용 현황 및 살포형태 등을 조사하고, 농약에 의한 질병이 발생할 수 있는 정보를 확인하는 것입니다. 답하신 개인정보는 공공기관의 개인정보보호에 관한 법률등에 의거 농촌진흥청에서 엄격히 관리되며, 향후 질환 발생 확인을 위한 목적이외에는 사용하지 않습니다. 조사에 참여해 주셔서 대단히 감사합니다.**  **1. 본인은 설문조사의 취지와 과정에 대한 설명을 듣고 자유로운 의사에 따라 응답하였습니다. ( ) 예, ( ) 아니오**  **2. 본인은 질환의 발생과 사망여부를 확인하기 위한 목적으로 공공자료(통계청, 국민건강보험공단, 국립암센터 등)를 연계하여 질병 발생여부를 확인하는 것에 동의합니다.**  **( ) 예, ( ) 아니오**  **참여자 성명_______________, 서명_____________** | | | | | | | | | | | | | | | | | | | | |
|  | | | | | | | | | | | | | | | | | | | | |
| **성 명** |  | | | | | | | | | | | | | **성 별** | | | | | ****_1_ 남자 ****_0_ 여자 | |
| **주민등록번호** |  |  |  | |  |  |  | | **-** |  |  | |  |  |  | |  |  | **실제나이**  **(주민번호와 다른경우)** | 만 세 |
| **연락전화번호** |  | | | | | | | | | | | | | | | | | | | |
| **조사 일시** | **2012 년** | | | **월** | | | | **일** | | | | **조사자 성명** | | | |  | | | | |
|  |  | | |  | | | |  | | | |  | | | |  | | | | |
|  |  | | |  | | | |  | | | |  | | | |  | | | | |
|  |  | | |  | | | |  | | | |  | | | |  | | | | |

|  | | | | | | | | |
| --- | --- | --- | --- | --- | --- | --- | --- | --- |
|  | | | | | | | | |
| **1.** 귀하께서 현재 **거주하고 계시는 지역과 거주기간에** 대해서 여쭈어 보겠습니다. | | | | | | | | |
|  | | | | | | | | |
|  | **거주 기간** | | **거주지 주소** | | | | | |
| 현재 거주지 | 년 월부터 년 월까지 | | 특별시/광역시/도 시/군/구  읍/면/동 리 번지 | | | | | |
|  | | | | | | | | |
| **2.** 귀하는 **현재 농사를 짓고 계십니까?** | | | | | | | | |
| ****_0_ 아니오. 전혀 농사를 지은 적이 없습니다. | | **☞ Part 2. 건강영향 문항으로 이동하세요.** | | | | |  | |
|  | | | | | | | | |
| ****_1_ 현재는 아니지만 과거에 농사를 지은 적이 있습니다. (**2-1. 과거에 농사 지은 총 기간** 년) | | | | | | | | |
| \| **☞ Part 2. 건강영향 문항으로 이동하세요.** \| \| --- \| | | | | | | | | |
| ****_2_ 예. 현재 농사를 짓고 있습니다. (**2-2. 농업을 주업으로 시작한 나이는?** 만 세 | | | | | | 총 기간 년 | | |
|  | | | | | | | | |
| **3.** 귀하는 **현재 농약을 살포**하고 계십니까? | | | | | | | | |
| ****_0_ 아니오. 지금까지 전혀 농약을 살포한 적이 없습니다. | | | | **☞ Part 2. 건강영향 문항으로 이동하세요.** | | | |  |
| ****_1_ 현재는 아니지만 **과거에** 농약을 살포한 적이 있습니다. | | | (**3-1. 과거에 농약을 살포한 총 기간** 년) | | | | | |
|  | | |  | | **☞ Part 2. 건강영향 문항으로 이동하세요.** | | | |
|  | | | | | | | | |
| ****_2_ 농약을 직접 살포하지는 않지만 **농약 살포 작업자를 도와주고** 있습니다 (농약혼합, 약줄 잡기 등). | | | | | | | | |
| ****_3_ 예. **현재 농약을 직접 살포**하고 있습니다. | | | | | | | | |
| **3-2. 총 몇 년간** 농약을 살포하셨습니까? 년 | | | | | | | | |
| **3-3. 매년** 평균적으로 **며칠간** 농약을 살포하셨습니까? 1년 평균 일 | | | | | | | | |
| **3-4. 농약 살포 일에** 평균적으로 **몇시간** 정도를 살포하셨습니까? 하루 평균 시간 | | | | | | | | |
| **3-5.** 농약을 사용하실 때 **2개 이상의 농약을 혼합(조제)하여** 사용하십니까? | | | | | | | | |
| ****_0_ 아니오. | | | | | | | | |
| ****_1_ 예. (**3-6. 몇 종류의** 농약을 혼합하여 사용하십니까? 종류) | | | | | | | | |
| **3-7**. **처음** 농약을 사용하신 **나이**가 몇 살 이었습니까? 만 세 | | | | | | | | |

| **4.** 귀하께서 **지난 한해동안 직업적으로 재배한 작물 및 가축을 대상으로 사용한 개별 농약**에 대하여 여쭈어 보겠습니다.  작목별 **사용농약**과 **살포방법**은 **보기에 따라 번호를** 적어주세요. | | | | | | | | | |
| --- | --- | --- | --- | --- | --- | --- | --- | --- | --- |
| **구분** | **작목명** | **규모(평/두수)** | **농사기간** | **연간 살포 회수** | **1회당 살포시간** | **1회당 살포량 (L or Kg)** | **살포방법 (보기②)** | **농약을 처음으로 사용하신 것은 언제 입니까?** | **총 몇 년간 농약을 살포하셨습니까?** |
| **수도작** | 벼 |  | 년 - 년 |  |  |  |  | 년 |  |
| **노지 재배 작물** |  |  | 년 - 년 |  |  |  |  | 년 |  |
|  |  |  | 년 - 년 |  |  |  |  | 년 |  |
|  |  |  | 년 - 년 |  |  |  |  | 년 |  |
| **과수** |  |  | 년 - 년 |  |  |  |  | 년 |  |
|  |  |  | 년 - 년 |  |  |  |  | 년 |  |
|  |  |  | 년 - 년 |  |  |  |  | 년 |  |
| **시설 재배 작물** |  |  | 년 - 년 |  |  |  |  | 년 |  |
|  |  |  | 년 - 년 |  |  |  |  | 년 |  |
|  |  |  | 년 - 년 |  |  |  |  | 년 |  |
| **가축** |  |  | 년 - 년 |  |  |  |  | 년 |  |
|  |  |  | 년 - 년 |  |  |  |  | 년 |  |
|  |  |  | 년 - 년 |  |  |  |  | 년 |  |
| **농약살포방법 (보기②):** | | | | | | | | | |
| 1. 핸드 스프레이 (동력식) 2. 핸드 스프레이 (수동식) 3. 등짐형 분무기 (동력식) 4. 등짐형 분무기 (수동식) 5. 고압 분무기 (SS기)-캡 있음 | | | | | | | | | |
| 6. 고압 분무기 (SS기)-캡 없음 7. 연무기 8. 손으로 뿌림 (입제, 분제 등) 9. 종자 소독 (액에 담금) 10. 기타 (표에 직접 방법을 적어 주십시오.) | | | | | | | | | |

| **5.** **직업적 농약 살포 작업**과 관련하여 **아래 보호장구별 사용정도**를 표시해 주십시오(✔표). | | | | | | | | |
| --- | --- | --- | --- | --- | --- | --- | --- | --- |
| **보 호 장 구** | **사 용 빈 도** | | | | | | | |
|  | **거의 착용 안함** | **가끔 착용** | **거의 착용** | | | **항상 착용** | | |
| **(1) 방제복 상의** | ① | ② | ③ | | | ④ | | |
| **(2) 방제복 하의** | ① | ② | ③ | | | ④ | | |
| **(3) 모자** | ① | ② | ③ | | | ④ | | |
| **(4) 가스 마스크** | ① | ② | ③ | | | ④ | | |
| **(5) 고글** | ① | ② | ③ | | | ④ | | |
| **(6) 고무장갑** | ① | ② | ③ | | | ④ | | |
| **(7) 고무장화** | ① | ② | ③ | | | ④ | | |
| *** 면 마스크, 면장갑 등은 해당 안됨** | | | | | | | | |
|  | | | | | | | | |
| **6.** **직업적 농약 살포 작업**과 관련하여 **아래 항목별 준수여부**를 표시해 주십시오(✔표). | | | | | | | | |
| **준 수 사 항** | | | | **준 수 여 부** | | | | |
|  |  |  |  | **지키지 않는다** | **가끔씩**  **지킨다** | | **대체로**  **지킨다** | **반드시 지킨다** |
| **(1) 사용법과 용량을 설명대로 지킨다** | | | | ① | ② | | ③ | ④ |
| **(2) 농약을 희석할 때에도 마스크와 장갑을 착용한다** | | | | ① | ② | | ③ | ④ |
| **(3) 농약 살포 중엔 음주나 흡연을 하지 않는다** | | | | ① | ② | | ③ | ④ |
| **(4) 피곤할 때는 뿌리지 않는다** | | | | ① | ② | | ③ | ④ |
| **(5) 한낮에는 뿌리지 않는다** | | | | ① | ② | | ③ | ④ |
| **(6) 농약 살포 작업 후엔 곧바로 옷을 갈아 입는다** | | | | ① | ② | | ③ | ④ |
| **(7) 농약 살포 작업 후엔 비누로 목욕을 한다** | | | | ① | ② | | ③ | ④ |
| **(8) 농약 살포시 바람을 등지고 한다** | | | | ① | ② | | ③ | ④ |
| **(9) 한 시간 작업 후 10분 휴식을 한다** | | | | ① | ② | | ③ | ④ |
| **(10) 농약 작업 시 사용했던 장비를 수리하거나 세척할 때 마스크와 장갑을**  **착용한다** | | | | ① | ② | | ③ | ④ |

|  | | | | | | | | | |
| --- | --- | --- | --- | --- | --- | --- | --- | --- | --- |
|  | | | | | | | | | |
|  | | | | | | | | | |
| **1.** 아래 항목에 있는 **만성질환을 앓았거나 앓고 계신 경우** 해당 내용에 체크하여 주십시오(✔표). | | | | | | | | | |
|  | | | | | | | | | |
|  | **지금까지 앓은 적 있음** | | **최근 1년간 3개월 이상 앓았음** | | **의사에게 진단을 받았음** | | **의사에게 처음 진단 받은 시기** | **현재 치료여부** | |
|  | **아니오** | **예** | **아니오** | **예** | **아니오** | **예** |  | **아니오** | **예** |
|  |  |  |  |  |  |  |  |  |  |
| **A. 순환기계** |  |  |  |  |  |  |  |  |  |
| **(1) 고혈압** | ☐_0_ | ☐_1_ | ☐_0_ | ☐_1_ | ☐_0_ | ☐_1_ | 만 세 | ☐_0_ | ☐_1_ |
| **(2) 고지혈증** | ☐_0_ | ☐_1_ | ☐_0_ | ☐_1_ | ☐_0_ | ☐_1_ | 만 세 | ☐_0_ | ☐_1_ |
| **(3) 뇌졸중(중풍)** | ☐_0_ | ☐_1_ | ☐_0_ | ☐_1_ | ☐_0_ | ☐_1_ | 만 세 | ☐_0_ | ☐_1_ |
| **(4) 심근경색증** | ☐_0_ | ☐_1_ | ☐_0_ | ☐_1_ | ☐_0_ | ☐_1_ | 만 세 | ☐_0_ | ☐_1_ |
| **(5) 협심증** | ☐_0_ | ☐_1_ | ☐_0_ | ☐_1_ | ☐_0_ | ☐_1_ | 만 세 | ☐_0_ | ☐_1_ |
|  |  |  |  |  |  |  |  |  |  |
|  |  |  |  |  |  |  |  |  |  |
| **B. 근골격계** |  |  |  |  |  |  |  |  |  |
| **(6) 골관절염** | ☐_0_ | ☐_1_ | ☐_0_ | ☐_1_ | ☐_0_ | ☐_1_ | 만 세 | ☐_0_ | ☐_1_ |
| **(7) 류마티스성 관절염** | ☐_0_ | ☐_1_ | ☐_0_ | ☐_1_ | ☐_0_ | ☐_1_ | 만 세 | ☐_0_ | ☐_1_ |
| **(8) 골다공증** | ☐_0_ | ☐_1_ | ☐_0_ | ☐_1_ | ☐_0_ | ☐_1_ | 만 세 | ☐_0_ | ☐_1_ |
| **(9) 허리 디스크** | ☐_0_ | ☐_1_ | ☐_0_ | ☐_1_ | ☐_0_ | ☐_1_ | 만 세 | ☐_0_ | ☐_1_ |
|  | | | | | | | | | |
| **C. 호흡기계** | | | | | | | | | |
| **(10) 폐결핵** | ☐_0_ | ☐_1_ | ☐_0_ | ☐_1_ | ☐_0_ | ☐_1_ | 만 세 | ☐_0_ | ☐_1_ |
| **(11) 폐외결핵** | ☐_0_ | ☐_1_ | ☐_0_ | ☐_1_ | ☐_0_ | ☐_1_ | 만 세 | ☐_0_ | ☐_1_ |
| **(12) 천식** | ☐_0_ | ☐_1_ | ☐_0_ | ☐_1_ | ☐_0_ | ☐_1_ | 만 세 | ☐_0_ | ☐_1_ |
| **(13) 만성폐쇄성폐질환 (만성기관지염,폐기종)** | ☐_0_ | ☐_1_ | ☐_0_ | ☐_1_ | ☐_0_ | ☐_1_ | 만 세 | ☐_0_ | ☐_1_ |
| **(14) 부비동염** | ☐_0_ | ☐_1_ | ☐_0_ | ☐_1_ | ☐_0_ | ☐_1_ | 만 세 | ☐_0_ | ☐_1_ |
| **(15) 기관지확장증** | ☐_0_ | ☐_1_ | ☐_0_ | ☐_1_ | ☐_0_ | ☐_1_ | 만 세 | ☐_0_ | ☐_1_ |
| **(16) 알레르기성 비염** | ☐_0_ | ☐_1_ | ☐_0_ | ☐_1_ | ☐_0_ | ☐_1_ | 만 세 | ☐_0_ | ☐_1_ |
|  | | | | | | | | | |
| **D. 내분비 대사성 질환** | | | | | | | | | |
| **(17) 당뇨병** | ☐_0_ | ☐_1_ | ☐_0_ | ☐_1_ | ☐_0_ | ☐_1_ | 만 세 | ☐_0_ | ☐_1_ |
| **(18) 갑상선장애** | ☐_0_ | ☐_1_ | ☐_0_ | ☐_1_ | ☐_0_ | ☐_1_ | 만 세 | ☐_0_ | ☐_1_ |

|  | **지금까지 앓은 적 있음** | | **최근 1년간 3개월 이상 앓았음** | | **의사에게 진단을 받았음** | | **의사에게 처음 진단 받은 시기** | **현재 치료여부** | |
| --- | --- | --- | --- | --- | --- | --- | --- | --- | --- |
|  | **아니오** | **예** | **아니오** | **예** | **아니오** | **예** |  | **아니오** | **예** |
|  | | | | | | | | | |
| **E. 눈, 귀질환** | | | | | | | | | |
| **(19) 백내장** | ☐_0_ | ☐_1_ | ☐_0_ | ☐_1_ | ☐_0_ | ☐_1_ | 만 세 | ☐_0_ | ☐_1_ |
| **(20) 녹내장** | ☐_0_ | ☐_1_ | ☐_0_ | ☐_1_ | ☐_0_ | ☐_1_ | 만 세 | ☐_0_ | ☐_1_ |
| **(21) 망막변성** | ☐_0_ | ☐_1_ | ☐_0_ | ☐_1_ | ☐_0_ | ☐_1_ | 만 세 | ☐_0_ | ☐_1_ |
| **F. 암** | | | | | | | | | |
| **(22) 위암** | ☐_0_ | ☐_1_ | ☐_0_ | ☐_1_ | ☐_0_ | ☐_1_ | 만 세 | ☐_0_ | ☐_1_ |
| **(23) 간암** | ☐_0_ | ☐_1_ | ☐_0_ | ☐_1_ | ☐_0_ | ☐_1_ | 만 세 | ☐_0_ | ☐_1_ |
| **(24) 대장암** | ☐_0_ | ☐_1_ | ☐_0_ | ☐_1_ | ☐_0_ | ☐_1_ | 만 세 | ☐_0_ | ☐_1_ |
| **(25) 유방암** | ☐_0_ | ☐_1_ | ☐_0_ | ☐_1_ | ☐_0_ | ☐_1_ | 만 세 | ☐_0_ | ☐_1_ |
| **(26) 자궁경부암** | ☐_0_ | ☐_1_ | ☐_0_ | ☐_1_ | ☐_0_ | ☐_1_ | 만 세 | ☐_0_ | ☐_1_ |
| **(27) 폐암** | ☐_0_ | ☐_1_ | ☐_0_ | ☐_1_ | ☐_0_ | ☐_1_ | 만 세 | ☐_0_ | ☐_1_ |
| **(28) 기타 암**  **(암종: )** | ☐_0_ | ☐_1_ | ☐_0_ | ☐_1_ | ☐_0_ | ☐_1_ | 만 세 | ☐_0_ | ☐_1_ |
| **(29) 기타 암**  **(암종: )** | ☐_0_ | ☐_1_ | ☐_0_ | ☐_1_ | ☐_0_ | ☐_1_ | 만 세 | ☐_0_ | ☐_1_ |
|  | | | | | | | | | |
| **G. 소화기계** | | | | | | | | | |
| **(30) 위십이지장궤양** | ☐_0_ | ☐_1_ | ☐_0_ | ☐_1_ | ☐_0_ | ☐_1_ | 만 세 | ☐_0_ | ☐_1_ |
| **(31) B형 간염** | ☐_0_ | ☐_1_ | ☐_0_ | ☐_1_ | ☐_0_ | ☐_1_ | 만 세 | ☐_0_ | ☐_1_ |
| **(32) C형 간염** | ☐_0_ | ☐_1_ | ☐_0_ | ☐_1_ | ☐_0_ | ☐_1_ | 만 세 | ☐_0_ | ☐_1_ |
| **(33) 간경변증** | ☐_0_ | ☐_1_ | ☐_0_ | ☐_1_ | ☐_0_ | ☐_1_ | 만 세 | ☐_0_ | ☐_1_ |
|  | | | | | | | | | |
| **H. 기타질환** | | | | | | | | | |
| **(34) 우울증** | ☐_0_ | ☐_1_ | ☐_0_ | ☐_1_ | ☐_0_ | ☐_1_ | 만 세 | ☐_0_ | ☐_1_ |
| **(35) 아토피피부염** | ☐_0_ | ☐_1_ | ☐_0_ | ☐_1_ | ☐_0_ | ☐_1_ | 만 세 | ☐_0_ | ☐_1_ |
| **(36) 신부전** | ☐_0_ | ☐_1_ | ☐_0_ | ☐_1_ | ☐_0_ | ☐_1_ | 만 세 | ☐_0_ | ☐_1_ |

|  | | |
| --- | --- | --- |
| **2.** 귀하의 **직계 가족 (부모, 형제/자매, 자녀)께서 병∙의원에서 의사로부터** 다음 질환으로 진단 받았거나 그로 인해 사망한 분이 있습니까? 있는 경우에 체크해 주시고, 몇 분이 있으신지 적어 주십시오. | | |
|  | | |
| **질병명** | **없다** | **있다 (부모, 형제/자매, 자녀 중)** |
| **고혈압** | ☐_0_ | ☐_1_ ( 명) |
| **당뇨병** | ☐_0_ | ☐_1_ ( 명) |
| **협심증 또는 심근경색증** | ☐_0_ | ☐_1_ ( 명) |
| **뇌졸중[중풍]** | ☐_0_ | ☐_1_ ( 명) |
| **위암** | ☐_0_ | ☐_1_ ( 명) |
| **폐암** | ☐_0_ | ☐_1_ ( 명) |
| **유방암** | ☐_0_ | ☐_1_ ( 명) |
| **대장암** | ☐_0_ | ☐_1_ ( 명) |
| **간암** | ☐_0_ | ☐_1_ ( 명) |
| **자궁경부암** | ☐_0_ | ☐_1_ ( 명) |
| **담낭암** | ☐_0_ | ☐_1_ ( 명) |
| **갑상선암** | ☐_0_ | ☐_1_ ( 명) |
| **췌장암** | ☐_0_ | ☐_1_ ( 명) |
| **전립선암** | ☐_0_ | ☐_1_ ( 명) |
| **난소암** | ☐_0_ | ☐_1_ ( 명) |
| **비호지킨 림프종** | ☐_0_ | ☐_1_ ( 명) |
| **인두암** | ☐_0_ | ☐_1_ ( 명) |
| **후두암** | ☐_0_ | ☐_1_ ( 명) |
| **피부암** | ☐_0_ | ☐_1_ ( 명) |
| **고환암** | ☐_0_ | ☐_1_ ( 명) |
| **신장암** | ☐_0_ | ☐_1_ ( 명) |
| **뇌 및 중추신경계암** | ☐_0_ | ☐_1_ ( 명) |
| **다발성 골수종** | ☐_0_ | ☐_1_ ( 명) |
| **백혈병** | ☐_0_ | ☐_1_ ( 명) |
| **골수암** | ☐_0_ | ☐_1_ ( 명) |
| **결체조직 또는 기타 연조직 암** | ☐_0_ | ☐_1_ ( 명) |
| **침샘암** | ☐_0_ | ☐_1_ ( 명) |
| **기타 악성종양**  **( 암)** | ☐_0_ | ☐_1_ ( 명) |

|  | | |
| --- | --- | --- |
|  | | |
| **3.** 다음의 문항을 일고 귀하의 현재 상태에 해당하는 답에 표시해 주십시오(✔표). | | |
|  | | |
| **항 목** | **아니오** | **예** |
| **(1) 현재의 생활에 대체적으로 만족하십니까?** | ☐_0_ | ☐_1_ |
| **(2) 요즈음 들어 활동량이나 의욕이 많이 떨어지셨습니까?** | ☐_0_ | ☐_1_ |
| **(3) 자신이 헛되이 살고 있다고 느끼십니까?** | ☐_0_ | ☐_1_ |
| **(4) 셍활이 지루하게 느껴 질 때가 많습니까?** | ☐_0_ | ☐_1_ |
| **(5) 평소에 기분은 상쾌한 편이십니까?** | ☐_0_ | ☐_1_ |
| **(6) 자신에게 불길한 일이 닥칠 것 같아 불안하십니까?** | ☐_0_ | ☐_1_ |
| **(7) 대체로 마음이 즐거운 편이십니까?** | ☐_0_ | ☐_1_ |
| **(8) 절망적이라는 느낌이 자주 드십니까?** | ☐_0_ | ☐_1_ |
| **(9) 바깥에 나가기가 싫고 집에만 있고 싶으십니까?** | ☐_0_ | ☐_1_ |
| **(10) 비슷한 나이의 다른 노인들보다 기억력이 더 나쁘다고 느끼십니까?** | ☐_0_ | ☐_1_ |
| **(11) 현재 살아 있다는 것이 즐겁게 생각되십니까?** | ☐_0_ | ☐_1_ |
| **(12) 지금의 내 자신이 아무 쓸모없는 사람이라고 느끼십니까?** | ☐_0_ | ☐_1_ |
| **(13) 기력이 좋은 편이십니까?** | ☐_0_ | ☐_1_ |
| **(14) 지금 자신의 처지가 아무런 희망도 없다고 느끼십니까?** | ☐_0_ | ☐_1_ |
| **(15) 자신이 다른 사람들의 처지보다 더 못하다고 생각하십니까?** | ☐_0_ | ☐_1_ |
|  | | |
| **3-1. 최근 1년 동안** 죽고 싶다는 생각을 해 본 적이 있습니까? | | |
| ****_0_ 아니오 | | |
| ****_1_ 예 | | |
| **3-2. 최근 1년 동안** 실제로 자살시도를 해 본 적이 있습니까? | | |
| ****_0_ 아니오 | | |
| ****_1_ 예 | | |

|  | | | | | | | | |
| --- | --- | --- | --- | --- | --- | --- | --- | --- |
|  | | | | | | | | |
| **4. 지난 1년간** 농약으로 인한 중독경험이 있으셨습니까? | | | | | | | | |
| ****_0_ 아니오 | | | | | | | | |
| ****_1_ 예 | | | | | | | | |
|  | | | | | | | | |
| **5. 4번 문항에 “예”** 라고 답하신 분만 작성해주십시오. | | | | | | | | |
| **발생 일자** | **농약명** | **중독**  **당시 상황^1)^** | **의도성^2)^** | | **치료 여부** | | **치료 받은 곳^3)^** | **본인부담**  **치료비용** |
| 월 |  |  |  | | ****_0_ 치료 받지 않았다. | |  |  |
|  |  |  |  |  | ****_1_ 외래치료를 받았다. (통원 기간: 일) | |  | 원 |
|  |  |  |  |  | ****_2_ 입원치료를 받았다. (입원 기간: 일) | |  | 원 |
| 월 |  |  |  | | ****_0_ 치료 받지 않았다. | |  |  |
|  |  |  |  |  | ****_1_ 외래치료를 받았다. (통원 기간: 일) | |  | 원 |
|  |  |  |  |  | ****_2_ 입원치료를 받았다. (입원 기간: 일) | |  | 원 |
| 월 |  |  |  | | ****_0_ 치료 받지 않았다. | |  |  |
|  |  |  |  |  | ****_1_ 외래치료를 받았다. (통원 기간: 일) | |  | 원 |
|  |  |  |  |  | ****_2_ 입원치료를 받았다. (입원 기간: 일) | |  | 원 |
| **보기:** | | | | | | | | |
| **1) 중독 당시 상황** | | ① 농약살포 후 | | ② 농약 혼합 및 조제 후 | | ③ 농약살포기계 세척 후 | | |
|  |  | ④ 농작업 이외 기타 상황 ( ) | | | | | | |
| **2) 의도성** | | ① 작업중 | | ② 복용 | | ③ 기타 ( ) | | |
| **3) 치료 받은 곳** | | ① 보건소(지소) | | ② 병∙의원 | | ③ 기타 ( ) | | |
